# Performance of discrete wavelet transform‐based method in the detection of influenza outbreaks in Iran: An ecological study

**DOI:** 10.1002/hsr2.1245

**Published:** 2023-05-03

**Authors:** Sara Minaeian, Yousef Alimohamadi, Babak Eshrati, Firooz Esmaeilzadeh

**Affiliations:** ^1^ Antimicrobial Resistance Research Center, Institute of Immunology & Infectious Diseases Iran University of Medical Sciences Tehran Iran; ^2^ Health Research Center, Life Style Institute Baqiyatallah University of Medical Sciences Tehran Iran; ^3^ Department of Social Medicine, Center for Preventive Medicine Iran University of Medical Sciences Tehran Iran; ^4^ Department of Public Health, School of Public Health Maragheh University of Medical Sciences Maragheh Iran

**Keywords:** DWT, influenza, Iran, outbreaks, performance

## Abstract

**Background and Aim:**

Timely detection of outbreaks is one of the main purposes of the health surveillance system. The presence of appropriate methods in the detection of outbreaks can have an important role in the timely detection of outbreaks. Because of the importance of this issue, this study aimed to assess the performance of discrete wavelet transform (DWT) based methods in detecting influenza outbreaks in Iran from January 2010 to January 2020.

**Methods:**

All registered influenza‐positive virus cases in Iran from January 2010 to January 2010 were obtained from the FluNet web base tool, the World Health Organization website. The combination method that includes DWT and Shewhart control chart was used in this study. All analyses were performed using MATLAB software version 2018a Stata software version 15.

**Results:**

The Mean ± SD and median of reported influenza cases from January 2010 to January 2020 was 36 ± 108 and four cases per week. The combination of the DWT and Shewhart control chart with *K* = 0.25 had the most sensitivity. The most specificity in the detection of nonoutbreak days was seen in the combination of DWT and Shewhart control chart with *K* = 1.5, *K* = 1.75, and *K* = 2, respectively. The combination of DWT and Shewhart control chart with *K* = 0.5 had the best performance in the detection of outbreaks (sensitivity = 0.64, specificity: 0.90, Youden index: 0.54, and area under the curve [AUC]: 0.77).

**Conclusion:**

The DWT‐based method in detecting influenza outbreaks has acceptable performance, but it is recommended that this method's performance be assessed in detecting outbreaks of other infectious diseases.

## 1 BACKGROUND

Globally, influenza is one of the main causes of morbidity and mortality.^1^ Despite the many actions to control and prevent influenza, this infection remained one of the main challenges for public health systems.^2^ The importance of this disease is because of its nature and high transmissibility, causing multiple epidemics and pandemics. By considering influenza history, influenza pandemics and pandemics are inevitable each year.^3^, ^4^ Influenza has imposed heavy burden on public health systems globally.^5^ According to studies, the influenza virus causes 250,000–500,000 deaths globally each year.^6^ These viruses are highly transmissible between the infected person to other suspect cases through respiratory drops, and an infected person can transmit the infection to 1.1–1.8 other individuals every 2–3 days.^7^

The timely detection and early response to outbreaks are one of the main purposes of public health surveillance systems in all parts of the world.^8^ To prevent, control, and reduce the burden of the disease, the use of appropriate methods to detect and timely health interventions can minimize the damage caused by the disease and reduce the incidence of morbidity and mortality.^9^, ^10^ So the timely detection of outbreaks can play an important role in decreasing the morbidity, mortality, and burden of many infectious diseases.^1^, ^11^ There are different methods to use for the timely detection of outbreaks.^12^, ^13^, ^14^, ^15^, ^16^ Each of the outbreak detection methods used different approaches to detecting outbreaks, and they have different performances in detecting outbreaks.^17^ The DWT is a beneficial method in signal denoising and anomaly detection.^18^ But the use of DWT in terms of outbreak detection is few. Because of a lack of use of DWT‐based methods in detecting influenza outbreaks in Iran, this study aimed to address the performance of DWT in detecting influenza outbreaks in Iran from January 2010 to January 2020.

## 2 METHODS

### 2.1 Data

All registered influenza‐positive virus cases in Iran from January 2010 to January 2010 were obtained from the FluNet web‐based tool, the World Health Organization website (http://www.who.int/influenza/gisrs_laboratory/flunet/en). Besides, the status of virus activity, including outbreak activity, was obtained from Flu Net and considered the gold standard of influenza outbreak occurrence. We used aggregate data of 19,079 cases of influenza‐positive viruses in the weekly count.

### 2.2 Outbreak detection method

The combination method that includes DWT and Shewhart control chart was used in this study. First, the influenza time series data decomposed to three‐level in this combination method using Haar wavelet. The level of decomposition is determined according to different factors such as the length of time series. The output of this step was to produce approximation and details coefficients for each level. After decomposition, all details coefficients and final approximation are monitored using the Shewhart control chart. By monitoring all levels using the Shewhart control chart, the weeks with amounts between the lower and upper control limit changed to zero, and Points outside this range were retained for the reconstruction of the signal. The reconstructed signal includes nonalarm week for outbreaks (weeks with zero value) and alarm week for outbreaks (weeks with nonzero value). The alarms are considered as on detected outbreak by the method.

### 2.3 Shewhart control chart

Shewhart control chart is one of the most common charts for detecting aberration from the normal trend. This chart has a baseline and upper and lower limits that are symmetrical with the baseline. The upper and lower control limit using this chart is calculated as follows:

Control limit: *µ*±*kσ*, where *µ* and *σ* are the mean and standard deviation of reported cases, a *k* is the desired coefficient in calculating the confidence limit. In this study, the *k* was considered: 0.25,0.5,0.75,1,1.25,1.5,1.75, and 2. If a point was outside the Control limit, that point is known as a deviation from the normal trend.

#### 2.3.1 Discrete wavelet transform

In this method, the understudied time series data were decomposed into several details and approximation coefficients using the wavelet functions. The result of the mentioned process is the production of approximate and detailed coefficients at the first level. Then, the approximation coefficient result from the previous step is decomposed for producing the approximation and details coefficients of level two. This process may continue for several stages considering the frequency and length of the data. After the decomposition of the original signal, all levels of detail coefficients and the last level of approximation coefficient are monitored by the Shewhart control chart. In this step, the points that are in each of the mentioned levels within the upper and lower limits of the Shewhart diagram were converted to zero and the other points were used to reconstruct the final signal. And finally, the final signal is used by the Shewhart chart to detect the deviation from the normal trend.^19^, ^20^

### 2.4 Assessment of the performance of the method

The performance of the understudy method was measured using sensitivity, specificity, false alarm rate, likelihood ratios, and area under the receiver operating characteristics (ROC) curve (AUC). Descriptive statistics, including mean, standard deviation (SD), median, minimum, and maximum of reported cases, were calculated using Microsoft Excel version 2010. The combined methods using DWT and Shewhart control chart were performed using MATLAB software version 2018a, and the ROC curve was plotted using the Stata software version 15.

## 3 RESULTS

The mean ± SD of reported influenza cases from January 2010 to January 2020 was 36 ± 108 cases per week. Also, the median of reported cases was four cases per week. Most influenza cases occurred in winter and autumn, respectively. The median (interquartile range) of reported cases in winter and autumn were 26(67‐11) and 12(66‐2) per week, respectively. Other important descriptive information is shown in Table 1.

**Table 1:** The descriptive statistics of influenza cases from January 2010 to January 2020.

Season	Median	Q1^a^	Q3^b^	Minimum	Maximum
Spring	2	1	9	0	35
Summer	0	0	1	0	26
Autumn	12	2	66	0	1017
Winter	26	11	67	0	388

^a^ Quartile 1.

^b^ Quartile 2.

**Figure 1:**
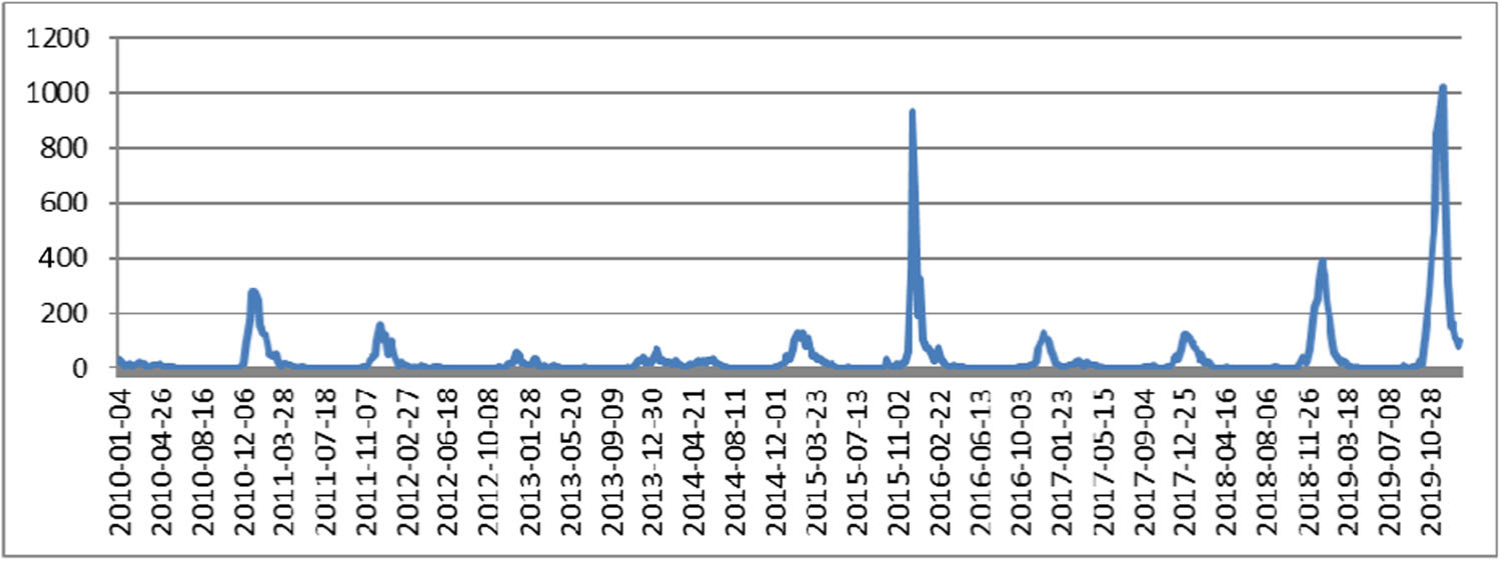
The line graph of the influenza cases from January 2010 to January 2020.

**Figure 2:**
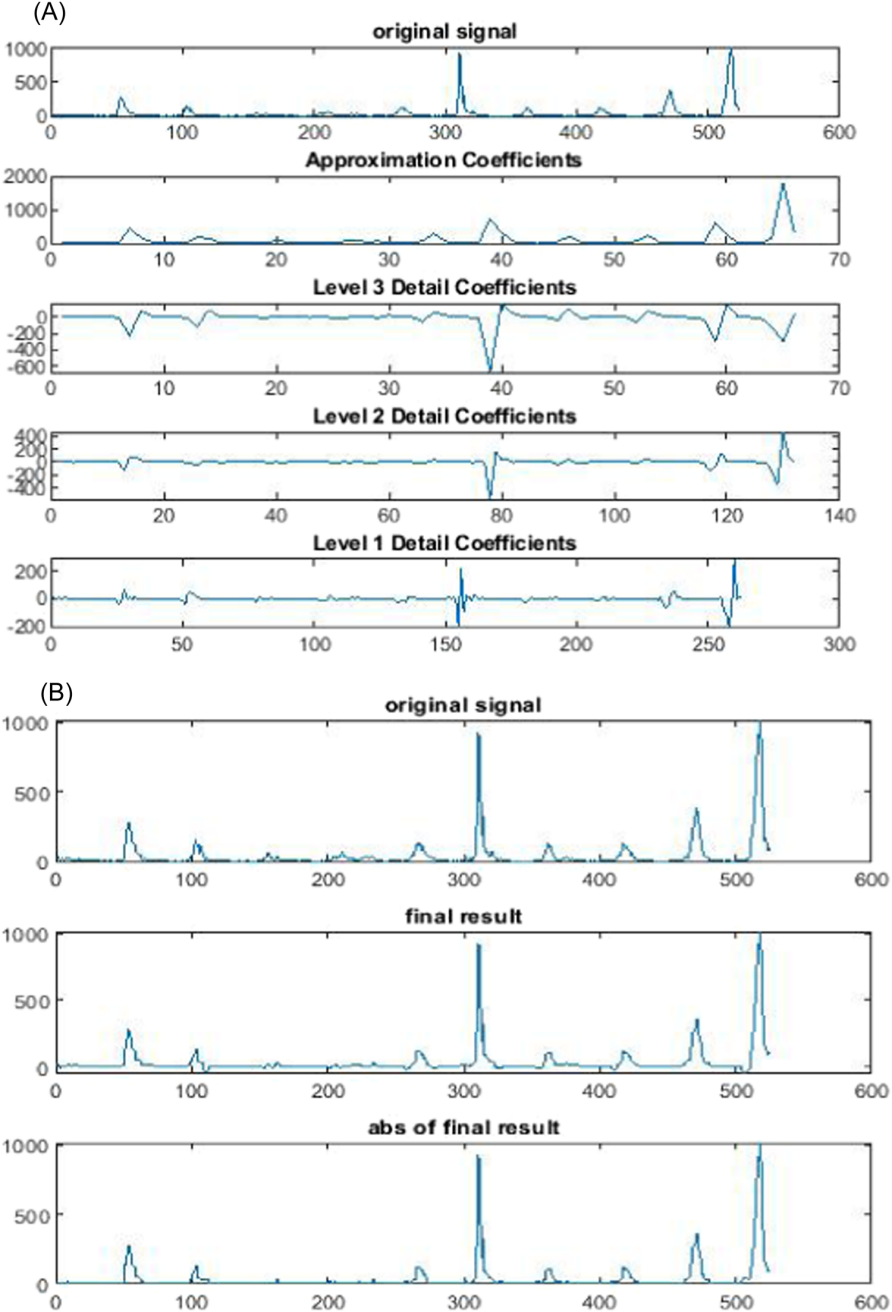
The three‐level discrete wavelet transform of influenza data (A) and detected outbreaks in reconstructed signal (B).

**Figure 3:**
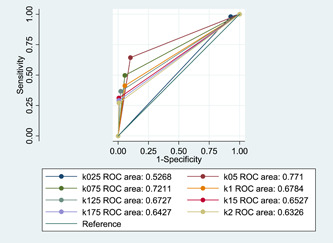
Area under the receiver operating characteristics (ROC) curve for a different amount of *K* in the discrete wavelet transform (DWT) and Shewhart control chart combination.

Regarding outbreak detection, the combination of DWT and Shewhart control chart with *K* = 0.25 had the most sensitivity. The most specificity in the detection of nonoutbreak days was seen in the combination of DWT and Shewhart control chart with *K* = 1.5, *K* = 1.75, and *K* = 2, respectively. The highest and the lowest False Alarm Rate were seen in *k* = 0.25 and *k* = 2, respectively. Also, the highest and lowest false‐negative rate was seen in *k* = 2 and *k* = 0.25, respectively. The highest and lowest Likelihood Ratio (LR)^+^ and LR^−^ were seen in a combination of DWT and Shewhart control chart with *K* = 0.25 and *K* = 2, respectively. Overall, the combination of DWT and Shewhart control chart with *K* = 0.5 had the best performance in the detection of outbreaks (sensitivity = 0.64, specificity: 0.90, Youden: 0.54, and area under the curve [AUC]: 0.77). Other important information is shown in Table 2 and Figures 1, 2, and 3.

**Table 2:** Performance combination of DWT and Shewhart control chart with different amounts of *K*.

Algorithm	Sensitivity	Specificity	False alarm rate	False negative	LR +	LR–	PPV	Youden index
*k* = 0.25	0.98 (0.96–1.00)	0.07 (0.05–0.10)	0.93 (0.90– 0.95)	0.02 (0.00–0.04)	1.06	0.27	0.39	0.05
*k* = 0.5	0.64 (0.58– 0.71)	0.90 (0.87–0.93)	0.10 (0.07– 0.13)	0.36 (0.29–0.42)	6.35	0.4	0.8	0.54
*k* = 0.75	0.50 (0.43–0.57)	0.94 (0.92–0.97)	0.06 (0.03–0.08)	0.50 (0.43–0.57)	9.01	0.53	0.85	0.44
*k* = 1	0.41 (0.34–0.48)	0.94 (0.92–0.97)	0.06 (0.03–0.08)	0.59 (0.52–0.66)	7.46	0.62	0.82	0.36
*k* = 1.25	0.37 (0.30–0.43)	0.98 (0.96–0.99)	0.02 (0.01–0.04)	0.63 (0.57–0.70)	17.08	0.65	0.91	0.35
*k* = 1.5	0.31 (0.25–0.38)	0.99 (0.99–1.00)	0.01 (0.00–0.01)	0.69 (0.62–0.75)	50.78	0.69	0.97	0.31
*k* = 1.75	0.29 (0.23–0.35)	0.99 (0.99–1.00)	0.01 (0.00–0.01)	0.71 (0.65–0.77)	47.51	0.71	0.97	0.29
*k* = 2	0.27 (0.21–0.33)	0.99 (0.99– 1.00)	0.01 (0.00–0.01)	0.73 (0.67–0.79)	44.23	0.73	0.96	0.27

Abbreviation: DWT, discrete wavelet transform.

## 4 DISCUSSION

The timely detection of influenza outbreaks can be very important for public health systems. By early detection of outbreaks, the number of incident cases and, consequently, the mortality due to this infection can be decreased significantly.^21^ The use of statistical methods is one of the beneficial tools for the timely detection of outbreaks and aberrations from the normal trend.^22^ So introducing an appropriate method with acceptable sensitivity can be very helpful for early warning of outbreaks by surveillance systems. Because of the importance of early detection of outbreaks, especially influenza outbreaks, this study evaluated DWT's performance in detecting outbreaks in Iran.

According to the results of this study, most influenza cases were reported in winter and autumn, respectively. This result is consistent with a similar study in other countries.^23^ This infection's high incidence and prevalence in some seasons may be related to virus activity, climate, temperature changes, and increased contact between infected cases and the suspect population. A study showed that most of the avian influenza outbreaks occurred during the cold seasons.^24^ It seems the pattern of humans (H1N1) and avian influenza (H5N1) has the same seasonal pattern.

The detection of influenza outbreaks in Iran and other countries was assessed by different methods.^1^, ^11^, ^13^, ^25^, ^26^, ^27^ A similar study about the application of discrete wavelet transform‐based methods in detecting outbreaks, especially influenza, is very rare. According to the results of this study, the highest sensitivity in the DWT‐based method was seen in *k*: 0.25 for the Shewhart control chart. It means the lowest amount of *k* for the Shewhart control chart gives the lowest threshold, so this caused an increase in sensitivity and decrease in specificity. The fewer amount of K leads to a few amount of alarm threshold, but the few thresholds can lead to an increase in the false alarm rate. According to our results, the DWT‐based method with *k*: 0.25 showed the highest false alarm rate, but a considerably higher amount of *k* can lead to a decrease in the false alarm rate. By considering our results, you can find the lowest false alarm rate in *K*: 2. So different amounts of *K* can lead to different results that should be considered in this method and other control charts.

In this study, the highest sensitivity of DWT based method was 0.98%. According to another study, the sensitivity of the exponential weighted moving average in detecting influenza outbreaks was 40%.^11^ So it seems the DWT has a better performance than exponentially weighted moving average in the detection of outbreaks days.

According to the result of this study, the DWT‐based method with *K*: 0.5 for Shewhart upper control limit had the best performance in detecting outbreaks days (Youden index: 0.54). So it seems the *k*: 0.5 in determining the upper control limit by the Shewhart chart can be beneficial in determining the alarm threshold level for influenza outbreaks.

## 5 LIMITATIONS

A limitation of this study must be noted. The number of studies comparing results was few, so the performance of the method must be evaluated in other studies.

## 6 CONCLUSION

The performance of DWT‐based method in detecting influenza outbreaks can be very helpful for health practitioners and policymakers. But it is recommended this method is used along with other methods because the combination of different outbreak detection methods can have better performance in detecting outbreaks, such as timely detection of outbreaks.

## AUTHOR CONTRIBUTIONS

**Sara Minaeian**: Conceptualization; methodology; supervision; writing—original draft; writing—review & editing. **Yousef Alimohamadi**: Formal analysis; methodology; writing—original draft; writing—review & editing. **Babak Eshrati**: Conceptualization; data curation; methodology; supervision; writing—original draft. **Firooz Esmaeilzadeh**: Writing—original draft; writing—review & editing.

## CONFLICT OF INTEREST STATEMENT

The authors declare no conflict of interest.

## ETHICS STATEMENT

This study was approved by the ethical committee of the Iran University of Medical Sciences with ID: IR.IUMS. REC.1399.1206.

## TRANSPARENCY STATEMENT

The lead author Yousef Alimohamadi affirms that this manuscript is an honest, accurate, and transparent account of the study being reported; that no important aspects of the study have been omitted; and that any discrepancies from the study as planned (and, if relevant, registered) have been explained.

## Data Availability

All data generated during this study are included in this published article.
